# Grid-based prediction of torsion angle probabilities of protein backbone and its application to discrimination of protein intrinsic disorder regions and selection of model structures

**DOI:** 10.1186/s12859-018-2031-7

**Published:** 2018-02-01

**Authors:** Jianzhao Gao, Yuedong Yang, Yaoqi Zhou

**Affiliations:** 1 School of Mathematical Sciences and LPMC, Nankai University, Tianjin, 300071 People’s Republic of China; 2 School of Data and Computer Science, Sun Yat-sen University, Guangzhou, 510000 People’s Republic of China; 3 Institute for Glycomics and School of Information and Communication Technology, Griffith University, Parklands Dr, Southport, QLD 4222 Australia

**Keywords:** Torsion angle, Intrinsically disordered region, Model quality assessment, Deep learning neural network

## Abstract

**Background:**

Protein structure can be described by backbone torsion angles: rotational angles about the N-Cα bond (φ) and the Cα-C bond (ψ) or the angle between Cα_i-1_-Cα_i_-Cα_i + 1_ (θ) and the rotational angle about the Cα_i_-Cα_i + 1_ bond (τ). Thus, their accurate prediction is useful for structure prediction and model refinement. Early methods predicted torsion angles in a few discrete bins whereas most recent methods have focused on prediction of angles in real, continuous values. Real value prediction, however, is unable to provide the information on probabilities of predicted angles.

**Results:**

Here, we propose to predict angles in fine grids of 5**°** by using deep learning neural networks. We found that this grid-based technique can yield 2–6% higher accuracy in predicting angles in the same 5**°** bin than existing prediction techniques compared. We further demonstrate the usefulness of predicted probabilities at given angle bins in discrimination of intrinsically disorder regions and in selection of protein models.

**Conclusions:**

The proposed method may be useful for characterizing protein structure and disorder. The method is available at http://sparks-lab.org/server/SPIDER2/ as a part of SPIDER2 package.

**Electronic supplementary material:**

The online version of this article (10.1186/s12859-018-2031-7) contains supplementary material, which is available to authorized users.

## Background

One of the most important sub problems of protein structure prediction is prediction of protein backbone secondary structure from sequences. Despite of the long history, the field of secondary structure prediction continues to flourish as the accuracy of three-state prediction (helix, sheet, and coil) steadily improves to 82–84% [1] because of larger sequence and structural databases [2–5] and more sophisticated deep learning neural networks [6, 7].

Instead of multi-state secondary structure, backbone structure of proteins can be more accurately described by continuous dihedral or rotational angles about the N-Cα bond (φ), the Cα-C bond (ψ) for single residues. A number of methods have been developed for prediction of angles in discrete states [8–11] or continuous values [6, 12–17]. For example, ANGLOR [15] employs neural networks and support vector machine to predict φ and ψ separately. TANGLE [16] utilizes a two-level support vector regression to predict backbone torsion angles (φ, ψ) from amino acid sequences. Li et al. [17] predicted protein torsion angles using four deep learning architectures, including deep neural network (DNN), deep restricted Boltzmann machine (DRBN), deep recurrent neural network (DRNN) and deep recurrent restricted Boltzmann machine (DReRBM). Most recently, Heffernan et al. [18] employed long short-term memory bidirectional recurrent neural networks that allows capture of nonlocal interactions and yielded the highest reported accuracy in angle prediction. Most recent review on torsion angle prediction can be found in [19]. Predicted angles have been proven useful in fold recognition [20, 21] and fragment-based [22] or fragment-free structure prediction [23]. A complementary description of backbone structure is to employ the angle between Cα_i-1_-Cα_i_-Cα_i + 1_ (θ) and the rotational angle about the Cα_i_-Cα_i + 1_ bond (τ). Unlike single-residue representation of φ and ψ angles, these two Cα-atom-based angles involve 3–4 locally connected residues. Predicted Cα-atom-based angles have demonstrated their potential usefulness in model quality assessment and structure prediction [6, 24].

Continuous, real value prediction of angles has the advantage over prediction of a few states as it provides a high-resolution description of backbone and removes the arbitrariness of defining boundaries between discrete states. Real-value prediction is a regression problem and it does not provide a separate confidence measure for predicted values. By comparison, prediction of discrete states is a classification problem and predicted probability of each class can be employed as a confidence measure. A confidence measure is needed because it allows conformational sampling of all angle regions in different probabilities, rather than a single angle in real-value prediction [8]. In fact, lack of a confidence measure for real-value prediction limited the usefulness of predicted angles as restrains for three-dimensional structure prediction [23]. Moreover, an accurate prediction of angle probability may provide useful information of conformational flexibility and, in the extreme case, protein intrinsic disorder [25]. One approach is to develop a separate method for predicting errors in predicted angles [26]. A reasonable accuracy was demonstrated between predicted and actual errors in angles with a Spearman correlation coefficient at 0.6.

In this study, we obtained the confidence measure of predicted angles by going back to discrete prediction. Early study by Kang et al. [8] divided φ and ψ angles into equal size bins of 10°. More coarse-grained grids were employed in later studies such as 30° by Bystroff et al. [10] and 40° by Kuang et al. [11]. This work employed a more refined, near-continuous discretization (5**°** bin in angles). Moreover, unlike previous methods, which is limited to torsion angles φ and ψ, we also predict Cα-atom-based angles θ and τ with the same fine grids. By using the same training and test sets as SPIDER2 [6], this fine-grid-based prediction not only achieves significantly more accurate prediction in given angle bins than SPIDER2, SPIDER3 [18] and other techniques without iterative multi-neural-network training but also provides the probabilities of predicted angles that might be useful for protein disorder prediction, protein structure prediction, and model quality assessment.

## Methods

### Datasets

To facilitate comparison, the datasets for the training and test of SPIDER2 [6, 27] were employed here for training and testing the neural network models. The training and test datasets contain 4590 (TR4590) and 1199 proteins (TS1199), respectively. These proteins have sequence identity less than 25% among them and their X-ray resolutions are better than 2 Å. Furthermore, we obtained a dataset that contained annotated structured and unstructured (intrinsically disordered) regions of 329 proteins (SL329), which was used by [28, 29]. Disordered regions in SL329 were annotated by DisProt [30] and Remark 465 in PDB [31] structure. Here, we tested the assumption that intrinsically disordered regions have a broad distribution of torsion angles and thus higher entropy in probabilities of predicted angles than structured regions.

In addition, we obtained all top 1 server models of 72 proteins in critical assessment of structure prediction (CASP 11). The CASP11MOD set has a total of 3017 models. The sequence identity between CASP11MOD and training dataset (TR4590) is less than 30%. We characterized the local structural quality of each model by sequence-position-dependent S-score [32]. *S*_*i*_ *= 1/(1 + (d*_*i*_*/d*_*0*_*)*^*2*^, where *d*_*0*_ *= 3 Å*, *d*_*i*_ was the distance between the residue *i* in the model structure and the same residue in the native structure. The pairwise structural alignment was performed by SPalign [33]. This dataset was employed for testing the usefulness of probabilities of predicted angles for structure prediction and model quality assessment.

Another independent test set is Rosetta decoy sets. It contains 58 native crystal protein structures with 100 lowest scoring models per native structure using Rosetta de novo structure prediction algorithm followed by all-atom refinement and 20 crystal structures that have been refined in Rosetta.

All datasets can be found at URL: http://sparks-lab.org/download/yueyang/data/spiderbin-dataset.tgz.

### Deep neural-network architecture

The deep neural network implemented by Palm [34] was employed for prediction of discrete angles. Stacked sparse auto-encoder was utilized for initializing unsupervised weights with learning rate of 0.05, which were refined by standard backward propagation. There were three hidden layers, with 150 hidden neurons in each layer with learning rates at 1.0, 0.5, 0.2, and 0.05 for different layers.

### Input features

We have built two separate models. The first model (M1) employed 27 features for each amino acid residue and a window size of 13 with 6 amino acid residues at each side of the query residue. The input features for a given amino acid residue are seven representative amino acid properties and Position Specific Scoring Matrix (PSSM) generated by PSI-BLAST [35] with three iterations of searching against NR database with an E-value of 0.001 (20 features). The seven amino acid properties are steric parameter (graph shape index), hydrophobicity, volume, polarizability, isoelectric point, helix probability, and sheet probability as we have employed in SPIDER2 [6, 27] *.*

In the second model (M2), we employed PSSM plus the output of SPIDER2 as input features, which includes predicted secondary structures, probabilities for three types of secondary structure (3 features), relative solvent accessibility (RSA) (1 feature), cosine/sine functions of backbone φ and ψ angles and Cα-atom-based angle θ and rotational angle τ (2*4 = 8 features), contact numbers based on Cα and Cβ atoms (CNα and CNβ, 2 features), respectively, and up and down half-sphere exposures (HSE) based on the Cα-Cβ vector and the Cα-Cα vector (HSEβ-up, HSEβ-down, HSEα-up, and HSEα-down, 4 features), respectively. We also used a sliding window size of 7 (3 amino acids at each side of the query amino acid residue) to represent each residue. This leads to 266 input features for per residue. We did not employ seven amino acid properties in M2 because they were employed in SPIDER2 and a smaller window size for M2 was employed because SPIDER2 has already employed a window size of 17 for its prediction.

### Outputs

For this grid-based method, all backbone angles were divided in 5° bin. φ, ψ, and τ ranging from − 180° to 180° have 72 bins, and θ ranging from 0° to 180° have 36 bins. In training, the actual angles are coded as 1 for the designated bin and 0, otherwise. A total of 252 (72*3 + 36) output nodes were employed for four angles, which are predicted simultaneously.

### Training, test and performance evaluation

The neural network model was trained by ten-fold cross validation with TR4590 and independently tested by TS1199. In the ten-fold cross validation, the training dataset was randomly divided into ten subsets. Nine subsets were employed for training and the remaining one subset was for test. This process repeated ten times so that all subsets were employed for test. Since predicting the torsion angles with 5° bin is a multi-class classification problem, the performance of angle prediction was evaluated by the number of correctly predicted angle bins in the total number of residues. The angle bin with the highest predicted probability is the predicted angle bin.

## Results

### Performance comparison

Table 1 compares the accuracy of four angle bins from SPIDER2 and two models [without (M1) or with (M2) SPIDER2 as input] from this work. It indicates that both models achieved higher accuracies for four angles on both training dataset (TR4590) and test dataset (TS1199). For the test set, there are 2–5% absolute improvements even without SPIDER2 (M1) as input with the highest improvement in θ angle. Inputting SPIDER2 prediction (M2) yielded a small but statistically significant improvement in bin accuracy (*p* < 2.9e-09 for all four angles) with the best improvement in θ (2%) and τ (1%) angles. The overall accuracy is 37% for θ and 19–20% for rotational angles (φ, ψ, and τ). In the test set, we further compared our method to SPIDER3 [18] and ANGLOR [15] in addition to SPIDER2. As shown in the table, our grid-based methods (M1 and M2) are more accurate in getting angles within 5° bin (e.g. 19.2% by M1 versus 14.0% by ANGLOR and 15.6% by SPIDER3 in φ, 17.1% by M1 versus 5.5% by ANGLOR and 15.7% by SPIDER3 in ψ).

**Table 1:** Accuracy for four angles, 5° for each bin

Dataset	Method	φ (Top 5^c^)	ψ (Top 5^c^)	θ (Top 5^c^)	τ (Top 5^c^)
TR4590	SPIDER2^a^	0.166	0.162	0.318	0.161
	M1^b^	0.196(0.607)	0.179(0.583)	0.365(0.799)	0.174(0.504)
	M2^b^	0.203(0.636)	0.187(0.616)	0.379(0.828)	0.185(0.547)
TS1199	ANGLOR	0.141	0.055	NA	NA
	SPIDER2^a^	0.162	0.151	0.304	0.153
	SPIDER3^a^	0.156	0.157	0.325	0.162
	M1^b^	0.192(0.598)	0.171(0.567)	0.358(0.794)	0.171(0.497)
	M2 ^b^	0.196(0.615)	0.174(0.588)	0.367(0.810)	0.178(0.528)

^a^Predicted real angle values from SPIDER2/SPIDER3 were evaluated according to 5° bin. ^b^M1 and M2 are models without or with SPIDER2 as input, respectively. ^c^ The number in parentheses is the accuracy of matching the native angles to one of the top five predicted angle bins

One nice feature of the grid-based prediction is that it can provide top predicted angles to choose from, rather than, a single angle in real-value prediction. As Table 1 showed, if the accuracy is measured by matching the native angles to one of the top five predicted angle bins, the accuracy increases 32–42% to 50–80% over top 1 for M1 and 35–44% to 53–81% over top 1 for M2. M2 consistently improves over M1 by 2–3% for top 5 matches in all four angles.

For structure prediction, large angle errors are the biggest concern. The φ angles can be split into two states [0° to 150°] and [(150° to 180°) and (− 180° to 0°)] and the ψ angles into [− 100° to 60°] and [(− 180° to − 100°) and (60° to 180°)]. SPIDER2 achieved 96.6% and 86.8% for two-state prediction of φ and ψ, respectively. By comparison, M1 achieved 96.0% and 84.2%, M2 achieved 96.5% and 86.8%, respectively. Thus, the large-angle error is comparable to SPIDER2, in the absence of iterative training.

One interesting question is whether or not a smaller number of output nodes would improve the accuracy of prediction. Table 2 compares the performance of the methods trained by 10° and 5° bins, respectively. For the test set (TS1199), the differences in correctly predicted angle bins for the methods trained by different angle bins are small (~ 0.3–0.4%). Thus, we will mainly focus on the methods based on the 5° bin.

**Table 2:** Accuracy for four angles, 10° for each bin in TS1199

Method	φ	ψ	θ	τ
SPIDER2^a^	0.292	0.263	0.458	0.241
M2–5°^b^	0.337	0.297	0.516	0.274
M2–10°^c^	0.340	0.300	0.520	0.277

^a^Predicted real angle values from SPIDER2 were evaluated based on 10° bin. ^b^Trained with SPIDER2 input and 5° bin and evaluated by combining two neighboring 5° bin. ^c^ Trained with SPIDER2 input and 10° bin

### Feature contributions

In order to evaluate the contributions from various features, we separated all features in M2 into three groups: PSSM-based features (PSSM profile), angle-based features (cosine/sine of predicted φ, ψ, θ and τ), and structure-based features (predicted secondary structure probability, relative solvent accessibility, half-sphere exposure, and contact numbers). As shown in Table 3, the model with angle-based features achieved the highest overall accuracy in three feature groups, followed by structure-based features. When two types of features are employed, the model using angle-based and PSSM-based features has a higher accuracy than that angle-based plus structure-based features. The M2 model with all three feature groups yields the best overall accuracy of angle bins and accuracy of top5 match. The improvement is statistically significant (*p*-value < 9.9e-02 over the best two feature groups and *p*-value < 1.8e-07 over the best single feature group).

**Table 3:** Accuracy for four angles, 5° for each bin, using different combinations of features groups in M2 on training dataset TR4590 with 10-fold cross validation. The number in parentheses is the accuracy of matching the native angles to one of the top five predicted angle bins

Method	φ (Top 5)	ψ (Top 5)	θ (Top 5)	τ (Top 5)
Angles-based features(Angles)^a^	0.200(0.629)	0.183(0.608)	0.374(0.823)	0.180(0.542)
Structure-based features(Struct)^b^	0.193(0.602)	0.176(0.583)	0.363(0.804)	0.174(0.521)
PSSM-based features(PSSM)^c^	0.188(0.588)	0.168(0.555)	0.353(0.784)	0.167(0.493)
Angles+PSSM	0.202(0.633)	0.186(0.613)	0.377(0.826)	0.184(0.545)
Angles+Struct	0.201(0.632)	0.185(0.611)	0.376(0.825)	0.182(0.544)
PSSM+Struct	0.198(0.622)	0.183(0.603)	0.373(0.819)	0.180(0.534)
All features of M2 model	0.203(0.636)	0.187(0.616)	0.379(0.828)	0.185(0.547)

^a^predicted angle feature group (φ and ψ angles and Cα-atom-based angle θ and rotational angle τ). ^b^ Structure-based feature group: predicted secondary structure probability, relative solvent accessibility, half-sphere exposure, and contact numbers. ^c^ PSSM based feature group: the features from PSSM profile

### Discrimination of protein disordered regions

**Fig. 1:**
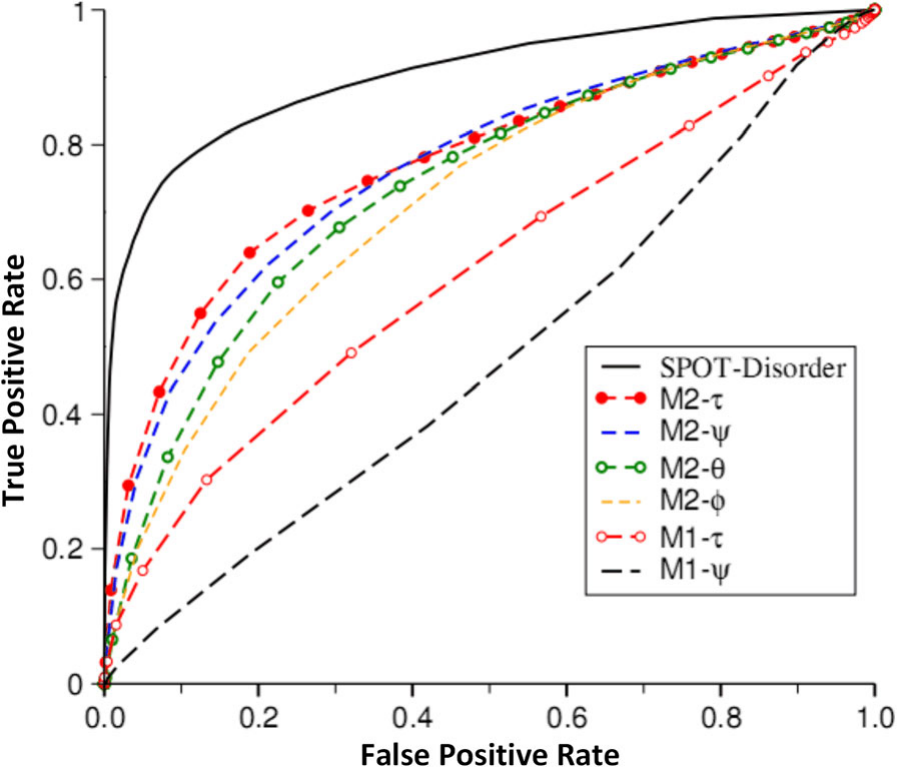
Receiver operating characteristic curve for disorder prediction given by a single feature from entropy of different angle probabilities predicted by M1 (PSSM + amino acid properties) and M2 (with SPIDER 2 as input), as compared to a deep-learning neural network based techniques SPOT-disorder employing multiple features

If predicted probabilities are actual representation of angle fluctuations, one would expect that angles in intrinsically disordered regions should have large fluctuation. In other words, predicted probabilities should be useful as a feature for predicting disordered regions. To test this concept, we compute the entropy *Entropy = −∑*_*i*_*P*_*i*_*log(P*_*i*_*)*. *P*_*i*_ is *i*-th angle bin probability. In order to evaluate the method based on area under the receiver operating characteristic curve (AUC), we normalized the entropy into (0, 1) by uniform distribution (Normalization has no effect on AUC). A window-based average of the entropy was employed as a single feature to predict protein disorder with the optimized window size of 21 residues at the query residue at the center. We found that the entropies based on angles predicted by M2 (with SPIDER2 as the input) are much better than those by M1, suggesting more accurately predicted probabilities by M2 (See Fig. 1). The former has AUC values between 0.55 and 0.64 by entropies based on different angles, compared to between 0.72 to 0.77 by M2. Entropy based on τ predicted by M2 has the highest discrimination capability with AUC = 0.77 between structured and intrinsically disordered regions. This is followed by M2-ψ, M2-θ and M2-φ**.** Better predictions by τ and θ than by ψ and φ are somewhat expected because the former angles are involving 3–4 residues and thus have a longer-range information than ψ and φ (single residue properties). This is consistent with the fact that structures built using predicted τ and θ are more accurate than those using predicted ψ and φ [14].

For comparison, we also listed one of the current-state-of-the-art techniques SPOT-disorder [36] which integrates multiple features by deep bidirectional long short-term memory recurrent neural networks. It achieves an AUC of 0.89 for the same dataset. Other methods such as DisEMBL (version 1.4) [37] and DISOPRED (version 3.16) [38] achieved AUC of 0.77 and 0.87, respectively. Thus, it is encouraging that a single feature from entropy based on angle probability fluctuation can achieve 0.77 for AUC. This indicates that the angle probability predicted by our method is physically reasonable as low and high entropies are linked to the regions with and without a well-defined structure, respectively.

### Model structure selection

Predicted angle probabilities can also be used to rank model structures. To do this, we calculate a pseudo-energy score for each model protein by defining PE-score=${\sum}_i\log \left({P}_i/{P}_i^0\right)$ where *P*_*i*_ is normalized predicted angle probability and ${P}_i^0$ is expected angle probability in the particular angle bin where each residue has positioned in the structural model. The performance of predicted angle probability for model ranking is measured by the Pearson correlation coefficient between PE-score and model accuracy (GDT_TS1 score) from the CASP11MOD dataset (See Methods). A high correlation indicates a simple relation between the overall quality of the model structure and the PE-score. Another measure is the model accuracy of the top 1 model. We compared the performance of PE-score with several established knowledge-based energy function (DFIRE [39], dDFIRE [40], and RWplus [41]).

**Fig. 2:**
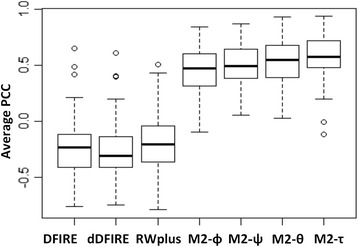
Average Pearson correlation coefficients for four angle based scores and statistical energy scores: DFIRE, dDFIRE and RWplus

Table 4 shows that the PE-scores based on all four angles have much higher correlation coefficients than commonly-used statistical energy scores (DFIRE, dDFIRE, and RWPlus) (positive correlations of 0.45–0.57 by M2 versus negative correlations of 0.20–0.27 by statistical energy functions). The model accuracy (measured by GDT scores) based on predicted top-1 ranked models ranges from 0.47 to 0.48 by PE-scores based on predicted angles, which are comparable to those given by statistical energy scores. Figure 2 Shows the boxplot of average PCCs for each target for different methods. It shows that M2-φ M2-ψ, M2-θ and M2-τ achieved higher average PCCs than absolute average PCCs of the DFIRE, dDFIRE and RWplus (*p*-value < 6.1e-06). For average GDT scores, there is no significant difference between the four angles and other three potential energy software as shown in Table 4 and Additional file 1: Figure S1.

**Table 4:** Performance in model selection according to average Pearson correlation coefficient (PCC) and average Global Distance Test (GDT) score of top 1 ranked models in the CASP11MOD dataset

Method	PCC ^a^ (median ^b^)	GDT
DFIRE	−0.24 (−0.23)	0.46
dDFIRE	−0.27(−0.31)	0.45
RWPlus	− 0.20(− 0.21)	0.47
M2 *- φ*	0.45(0.47)	**0.48**
M2 -ψ	0.49(0.49)	**0.48**
M2-θ	0.53(0.55)	0.47
M2-τ	**0.57(0.57)**	0.47

^a^Average 72 targets’ PCCs, ^b^Median of 72 targets’PCCs , and the best results were emphasized

**Fig. 3:**
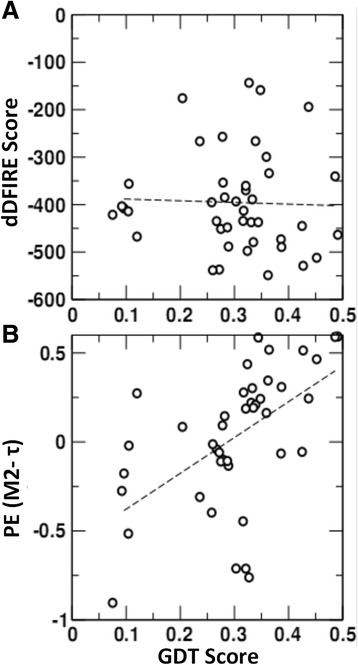
Scatter plot for quality scores and GDT score for target T0848. Dashed line is the regression line between quality scores and GDT scores. (A) dDFIRE energy score vs. GDT score, Pearson correlation coefficient is − 0.09 (B) M2-τ scores vs. GDT score, Pearson correlation coefficient is 0.55

**Fig. 4:**
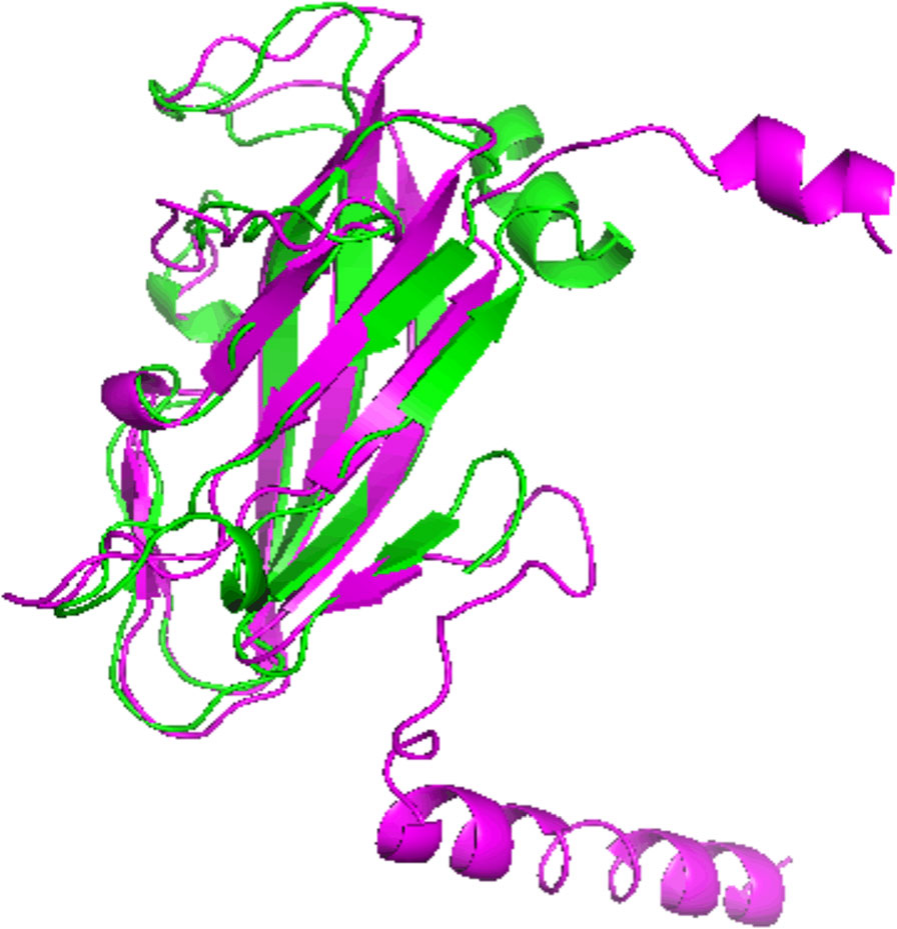
The alignment between the first domain of the selected model using M2-τ quality score in purple and the first domain of actual target T0848 structure (PDBID: 4R4G) in green

Take T0848 for example, T0848 is a hard target in CASP11. It contains two domains, T0848-D1:34–171; T0848-D2 172–354. Figure 3 shows that there is a higher correlation 0.55 between M2-τ quality scores and GDT scores than correlation − 0.09 of dDFIRE score. The selected model is BAKER-ROSETTASERVER_TS1 for T0848 using M2-τ quality score. (DFIRE, RWplus, M2-φ, M2-ψ, M2-θ scores, see Additional file 1: Figure S2-S6). Figure 4 visualizes the accuracy of the selected model by the alignment between the first domain of selected model and the first domain of actual target T0848 (PDBID: 4R4G).

To further test model selection, Table 5 shows the performance of our methods for the Rosetta decoy set. M2 method achieved average PCC of 0.43~ 0.53 and GDT scores of 0.66–0.72. Again, M2-τ has the best performance. For this specific dataset, the performance of predicted angle probabilities is comparable to the energy scores in terms of PCC or GDT scores.

**Table 5:** Performance in model selection according to average Pearson correlation coefficient (PCC) and average Global Distance Test (GDT) score of models in the Rosetta decoy set

Method	PCC ^a^ (median ^b^)	GDT
DFIRE	−0.53 (−0.71)	**0.72**
dDFIRE	−0.38(− 0.48)	0.59
RWPlus	−0.51(− 0.68)	0.70
M2 *- φ*	0.43(0.51)	0.66
M2 -ψ	0.48(0.65)	0.69
M2-θ	0.50(0.66)	**0.72**
M2-τ	**0.53(0.68)**	0.69

^a^Average 58 native structures’ PCCs, ^b^Median of 58 native structures’PCCs , and the best results were emphasized

## Discussion and Conclusion

In this work, we proposed a method to make grid-based angle prediction. Our methods achieved overall accuracy of 19%~ 38% on training dataset and 17%~ 37% on the test dataset with a grid of 5° angle bins, depending on specific angles. These accuracies are 2–6% higher than the real-value prediction of SPIDER2 or SPIDER3 for angles within 5°.

One advantage of using bins, rather than predicting real angle values is that using bins will yield the probability for predicted angles. We show that angle probability for a given bin is a very useful feature to identity the disordered region with AUC as high as 0.77 by M2 for a single feature based on predicted τ. The probability was also used as an energy score to score model structures and achieved better or comparable accuracy in model selection and higher or comparable average correlation coefficients between model accuracy and ranking scores as compared to statistical energy functions. The ability to characterize protein structure and disorder confirms that predicted probabilities are physically reasonable. It could be useful in real world applications of protein structure and disorder prediction as a complementary feature to other techniques. The software is available at: http://sparks-lab.org/server/SPIDER2/ as a part of SPIDER2 structure-property-prediction package.

## Additional file

Additional file 1: Supplementary Information for Grid-based Prediction of Torsion Angle Probabilities of Protein Backbone and Its Application to Discrimination of Protein Intrinsic Disorder Regions and Selection of Model Structures. **Figure S1:** Average GDT-TS scores of top 1 server models for different methods on the CASP11 dataset (CASP11MOD). **Figure S2:** Scatter plot for DFIRE energy scores and GDT-TS score for target T0848. Blue line is the regression line between DFIRE energy scores sand GDT-TS scores. Correlation coefficient is − 0.04. **Figure S3:** Scatter plot for RWplus energy scores and GDTTS score for target T0848. Blue line is the regression line between RWplus energy scores sand GDTTS scores. Correlation coefficient is − 0.03. **Figure S4:** Scatter plot for M2-φ energy scores and GDT-TS score for target T0848. Blue line is the regression line between M2-φ energy scores sand GDT-TS scores. Correlation coefficient is 0.42. **Figure S5:** Scatter plot for M2-ψ energy scores and GDT-TS score for target T0848. Blue line is the regression line between M2-ψ energy scores sand GDT-TS scores. Correlation coefficient is 0.49. **Figure S6:** Scatter plot for M2-θ energy scores and GDT-TS score for target T0848. Blue line is the regression line between M2-θ energy scores sand GDT-TS scores. Correlation coefficient is 0.46. (DOCX 221 kb)
