# Supplementary material for: Grid-based prediction of torsion angle probabilities of protein backbone and its application to discrimination of protein intrinsic disorder regions and selection of model structures
Source: BMC Bioinformatics. 2018 Feb 1;19:29. doi: 10.1186/s12859-018-2031-7 (PMC5796405; doi:10.1186/s12859-018-2031-7)
Supplement: Additional file 1: — Supplementary Information for Grid-based Prediction of Torsion Angle Probabilities of Protein Backbone and Its Application to Discrimination of Protein Intrinsic Disorder Regions and Selection of Model Structures. Figure S1: Average GDT-TS scores of top 1 server models for different methods on the CASP11 dataset (CASP11MOD). Figure S2: Scatter plot for DFIRE energy scores and GDT-TS score for target T0848. Blue line is the regression line between DFIRE energy scores sand GDT-TS scores. Correlation coefficient is − 0.04. Figure S3: Scatter plot for RWplus energy scores and GDTTS score for target T0848. Blue line is the regression line between RWplus energy scores sand GDTTS scores. Correlation coefficient is − 0.03. Figure S4: Scatter plot for M2-φ energy scores and GDT-TS score for target T0848. Blue line is the regression line between M2-φ energy scores sand GDT-TS scores. Correlation coefficient is 0.42. Figure S5: Scatter plot for M2-ψ energy scores and GDT-TS score for target T0848. Blue line is the regression line between M2-ψ energy scores sand GDT-TS scores. Correlation coefficient is 0.49. Figure S6: Scatter plot for M2-θ energy scores and GDT-TS score for target T0848. Blue line is the regression line between M2-θ energy scores sand GDT-TS scores. Correlation coefficient is 0.46. (DOCX 221 kb) [file 12859_2018_2031_MOESM1_ESM.docx]

Grid-based prediction of torsion angle probabilities of protein backbone and its application to discrimination of protein intrinsic disorder regions and selection of model structures

Jianzhao Gao^1^, Yuedong Yang^2,*^ and Yaoqi Zhou^3,*^

^1^School of Mathematical Sciences and LPMC, Nankai University, Tianjin, 300071, People’s Republic of China,

^2^School of Data and Computer Science, Sun Yat-sen University, Guangzhou, 510000, People’s Republic of China;

^3^Institute for Glycomics and School of Information and Communication Technology, Griffith University, Parklands Dr., Southport, QLD 4222, Australia

* To whom correspondence should be addressed.

Yuedong Yang* yangyd25@mail.sysu.edu.cn

Yaoqi Zhou* yaoqi.zhou@griffith.edu.au

# Supplemental Figures





**Figure S1:** Average GDT-TS scores of top 1 server models for different methods on the CASP11 dataset (CASP11MOD).


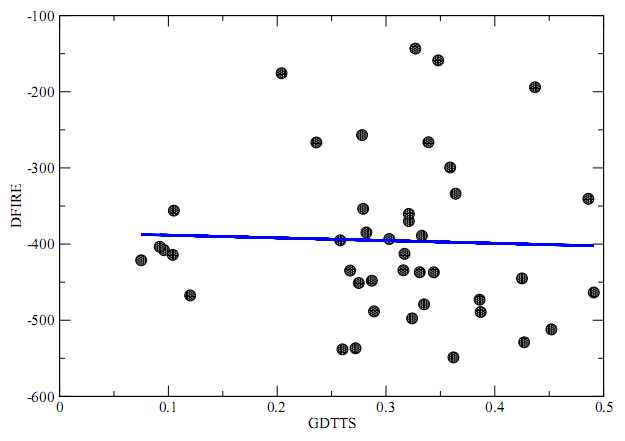


**Figure S2:** Scatter plot for DFIRE energy scores and GDT-TS score for target T0848. Blue line is the regression line between DFIRE energy scores sand GDT-TS scores. Correlation coefficient is -0.04.


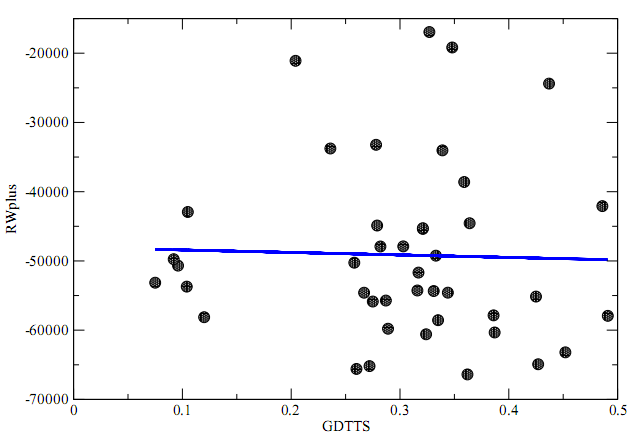


**Figure S3:** Scatter plot for RWplus energy scores and GDTTS score for target T0848. Blue line is the regression line between RWplus energy scores sand GDTTS scores. Correlation coefficient is -0.03.


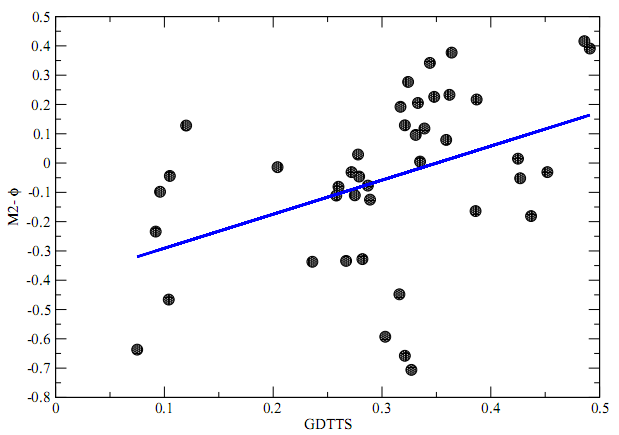


**Figure S4:** Scatter plot for M2-φ energy scores and GDT-TS score for target T0848. Blue line is the regression line between M2-φ energy scores sand GDT-TS scores. Correlation coefficient is 0.42.


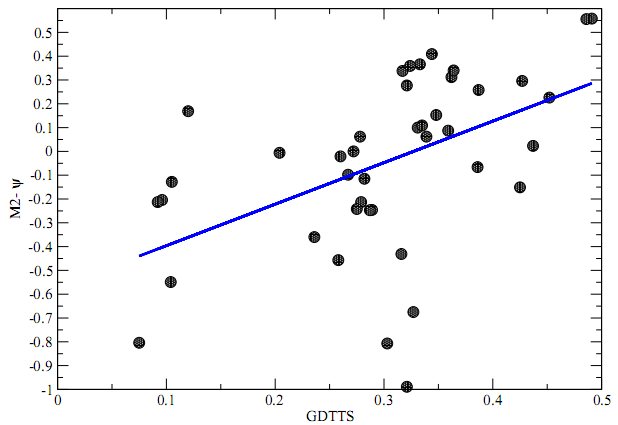


**Figure S5:** Scatter plot for M2-ψ energy scores and GDT-TS score for target T0848. Blue line is the regression line between M2-ψ energy scores sand GDT-TS scores. Correlation coefficient is 0.49.


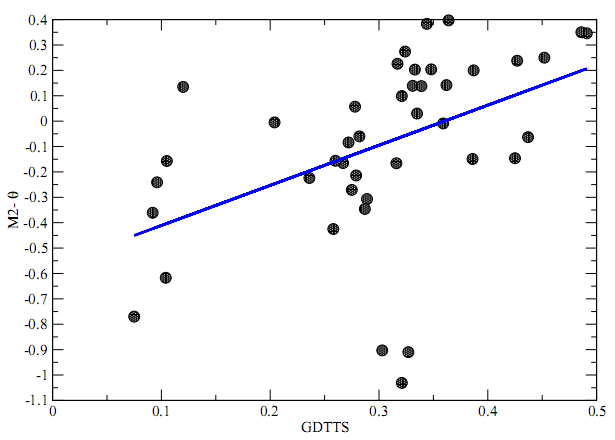


**Figure S6:** Scatter plot for M2-θ energy scores and GDT-TS score for target T0848. Blue line is the regression line between M2-θ energy scores sand GDT-TS scores. Correlation coefficient is 0.46.
